# *Saccharomyces cerevisiae* Yeast-Based Supplementation as a Galactagogue in Breastfeeding Women? A Review of Evidence from Animal and Human Studies

**DOI:** 10.3390/nu13030727

**Published:** 2021-02-25

**Authors:** Lili Lily Jia, Louise Brough, Janet Louise Weber

**Affiliations:** School of Food and Advanced Technology, College of Sciences, Massey University, Palmerston North 4442, New Zealand; L.Brough@massey.ac.nz (L.B.); J.L.Weber@massey.ac.nz (J.L.W.)

**Keywords:** *Saccharomyces cerevisiae*, brewer’s yeast, nutritional yeast, supplement, breastfeeding, breast milk, human milk, milk production, galactagogue

## Abstract

Perceived insufficient milk production (PIM) adversely affects breastfeeding duration. Women sometimes use galactagogues with the intent to increase breast milk production and support lactation. *Saccharomyces cerevisiae* yeast-based supplement (SCYS) is an inactive form of *Saccharomyces cerevisiae* yeast (SCY) either obtained from the fermentation process or grown on molasses. Anecdotal evidence suggests SCYS is a galactagogue. SCYS is promoted on the internet as a galactagogue in various forms and doses. Dietary supplementation with SCYS during gestation and lactation significantly increases milk yield in ruminants. No human study has evaluated efficacy of SCYS as a galactagogue. SCYS is rich in B vitamins, beta-glucan, mannan oligosaccharides and bioavailable chromium; these may impact breast milk production or composition, thus may alleviate PIM. The safety of taking SCYS during lactation is not well studied. Studies have reported contamination of SCYS with ochratoxin A (OTA) as well as minor side effects from SCYS. Studies are needed to evaluate the efficacy of SCYS on breast milk production and composition and to assess the safety of taking SCYS during lactation in humans.

## 1. Introduction

Perceived insufficient milk production (PIM) is a worldwide problem affecting breastfeeding duration [1,2,3,4,5,6]. Globally, approximately 35% of women who stopped breastfeeding before 4 months postpartum reported PIM as the primary reason for discontinuation [5].

Many factors can affect women’s perceptions of milk production. For example, milk production may actually be low due to some physical problems or medications which can suppress hormone release related to milk production [7]. In addition, lactation problems or difficulties with positioning and latching may decrease stimulation of the breast or decrease milk removal, which adversely influences milk production [8]. Furthermore, postnatal distress (depression, anxiety and stress), which influences oxytocin secretion, can also result in decreased milk production [8]. Maternal diet has limited impact on breast milk production. Maternal nutrition status, maternal body composition and maternal energy intake are not associated with breast milk volume [9]. Food supplementation to address malnutrition or maternal energy restriction has little effect on milk volume [10,11]. Maternal intake of some nutrients influences their respective concentration in breast milk [12,13].

Women may also incorrectly perceive their milk supply to be insufficient. For instance, some women may interpret their infants’ unsatisfied, unsettled or crying behaviour as meaning inadequate milk [5,14,15,16]. Whereas others may perceive that their milk looks watery, or that they have empty/soft breasts, or that the infant has frequent or long feeds and they mistake these as signs of the lack of quality or quantity of milk [15,17]. Good lactation management and support can enable women to overcome these situations and continue to breastfeed successfully. However, if PIM results in the introduction of supplementary feeding, this can reduce milk production and may ultimately result in the cessation of breastfeeding [17].

Women’s perception of milk supply may be influenced by health professionals, family, and friends. If these people questioned the mother’s milk supply, the mother would perceive her milk supply as inadequate [18].

To address PIM, women sometimes use galactagogues, which are medications, herbs, supplements and foods, to increase breast milk production and support lactation [19,20]. *Saccharomyces cerevisiae* yeast-based supplement (SCYS) is one galactagogue used by breastfeeding women [21,22], and some lactation specialists recommend SCYS based on anecdotal evidence of efficacy [19,23]. To date, no professional body has endorsed the use of SCYS as a galactagogue and there are no recommendations or guidelines for use during lactation.

*Saccharomyces cerevisiae* yeast (SCY) includes thousands of strains that have a long history of use in brewing and baking [24]. Strains used for brewing ale are named brewer’s yeast [25], whereas baker’s yeast strains provide the leavening to make bread [25]. In the brewing industry, “brewer’s yeast” is the common name for all types of yeast used for brewing beer. *Saccharomyces cerevisiae* is used to produce top fermenting beer, that is, ale [12]. In this review, brewer’s yeast only refers to *Saccharomyces cerevisiae*. SCYS, the inactive form of SCY (dead yeast cells without the fermentation or leavening property), is popular as a dietary supplement for humans. It is promoted as containing high concentrations of protein, some B vitamins and minerals, as well as beta-glucan and mannan oligosaccharides (MOS) from yeast cell walls [24,26]. Research investigating health benefits of SCYS for human participants mainly focuses on two areas. First, SCYS (i.e., brewer’s yeast) contains organic chromium with better absorption compared to inorganic chromium [27]. Thus, brewer’s yeast has been used for decades in research evaluating the effect of chromium supplementation on fasting plasma glucose, lipid metabolism and blood pressure in diabetic patients [28,29,30]. Second, beta-glucan derived from SCY cell walls modulates the innate immune system, and the effects of yeast beta-glucan supplementation on upper respiratory tract infection and allergy symptoms have been evaluated in a few trials [31,32]. The influence of SCYS on human lactation has not yet been investigated.

SCYS products are widely available as tablets, powders, flakes and in liquid form [26,33]. These products are from three different processes: spent yeast from brewing, yeast from fermentation with malted barley, and yeast grown on molasses. Yeast cultivated on malted barley is the same as brewing with the difference that the yeast is the product, not beer. Thus, products from the first two sources usually have a bitter taste from the fermentation [33,34], and they must be debittered and washed prior to drying [34]. The third source, SCY is grown on molasses enriched by additional nutrients such as minerals and B vitamins under aerobic conditions; the nutrients in the resultant SCYS depend on the composition of molasses [33].

SCYS is approved as a food additive for human consumption by the US Food and Drug Administration (USDA) [35]; however, there is no standard or regulation regarding the product names, sources or dosage of SCYS.

In the literature, SCYS used for diabetic participants is commonly named “brewer’s yeast”, but information of the strain and product source or process is generally absent [28,29,36]. However, in marketing material, SCYSs are defined by their method of production: SCYS from the fermentation processes is commonly named as “brewer’s yeast” or “nutritional brewer’s yeast”; SCYS grown on molasses is named as “nutritional yeast” or “brewer’s type yeast” [26,33,37]. However, it is not reported in the literature if the different designations imply variation of the nutrition composition of SCYS.

Although used by breastfeeding women, it is unknown how or if SCYS influences breast milk production. This review aims to evaluate evidence of the effect of SCYS on milk production in other mammals, to propose possible mechanisms that human milk production could be influenced, and to review the safety of using SCYS during lactation. In addition to reviewing the literature, we used Google search engine to obtain an overview of SCYS on the market and the anecdotal recommendations posted for breastfeeding women.

## 2. An Overview of Information of *Saccharomyces cerevisiae* Yeast-Based Supplement on the Internet

Alianmoghaddam et al. suggested Google is breastfeeding women’s main source of information for lactation problems [38]. A Google search of advertisements for SCYS products sold online highlighted that product designation was confusing within the SCYS market, especially the term “brewer’s yeast”. We used “brewer’s yeast” or “nutritional yeast” as keywords for searching. We chose the first 10 products without duplicates to show the variation of product name, source and daily dose (Table 1). Some of the products named “brewer’s yeast” were reported to be grown on molasses. Some of the products explicitly indicated the medium or the production process, but others provided no information. Furthermore, some products provided information of yeast species, but the information of strains was missing from all products. Although it is assumed that products from fermentation processes use brewer’s yeast strains, there is no denying the possibility that the products grown on molasses may use baker’s yeast strains. The information presented in Table 1 suggests a range of “brewer’s yeast” products aimed at breastfeeding women are available for purchase online.

Furthermore, the reported nutrient concentrations varied among products. Only five of the products from Table 1 had nutrition information available online. Table 2 shows the concentrations of B vitamins and minerals of these products. Vitamin concentrations in SCYS vary by over 100-fold. Nutrient concentrations of products with the same name (e.g., brewer’s yeast) may also differ from each other.

The difference in nutrient content between different SCYS products is caused by several factors. First, different strains grown in the same medium have different growth patterns and biomass [40]. For example, brewer’s yeast strains contain higher concentrations of chromium compared to baker’s yeast strains [24]. Second, the composition of spent yeast varies between different fermentation processes due to the growth conditions, material of the brewing plant and yeast recycling [24,34]. Third, the growing medium has significant influence. For example, yeast grown on enriched molasses has higher concentrations of some B vitamins and minerals [37], and cultures enriched with chromium salts or selenium salts are used to produce chromium yeast or selenium yeast [41,42,43]. Lastly, as shown in Table 1, extra B vitamins may be added to the final product, which leads to a much higher B vitamin concentration than unfortified products grown on unenriched medium (Table 2).

Although SCYS is not endorsed as a galactagogue by any professional organisation there are many references to this use of it on the internet. We searched “brewer’s yeast”, “nutritional yeast” or “*Saccharomyces cerevisiae*” with “breast milk”, “milk production” or “milk supply” on Google and reviewed the first 50 results of each combination. After removing duplicates, we chose the articles and recipes which either indicated the author’s qualification in health or breastfeeding or was provided by breastfeeding advocates (i.e., doula and breast pump manufacturer) as shown in Table 3. The daily dose of SCYS recommended in these articles and recipes is below 5 g if suggested in tablet form but is as high as 30 g in the suggestion of adding three tablespoons of powder to a drink. It is more difficult to evaluate the daily intake if SCYS is added to lactation cookies, because most recipes only provide the amount to add to a batch, but the daily dose depends on the cookie size and numbers of cookies eaten per day. Furthermore, one online article insisted that brewer’s yeast was the only effective yeast to increase breast milk production [44], whereas another stated that both brewer’s yeast and nutritional yeast were *Saccharomyces cerevisiae* yeast, and that both provided B vitamins and so were interchangeable [45]. The lack of consistent advice and the variable nutrient composition of the commercial products has efficacy and safety implications.

## 3. Using *Saccharomyces Cerevisiae* Yeast as a Galactagogue in Ruminants and Non-Ruminants

SCY products are widely used as dietary supplements to increase milk yield in ruminants [46,47,48,49,50,51,52,53,54,55,56,57,58] and to improve the reproductive performance of sows and growth of offspring [59,60,61,62,63,64,65]. These products include active SCY, inactive SCY (SCYS) and yeast cell wall products (YCWPs), such as beta-glucan, MOS or combined products. The benefit of active SCY on milk production is attributed mainly to probiotic properties, which improve nutrient digestibility and metabolism [46,59,60,61]. However, use of live yeast is irrelevant in humans because dietary supplements are in the form of SCYS (i.e., inactive cells) [43], so the effect of active SCY will not be further discussed in this review.

We reviewed studies on supplementation of lactating animals using SCYS and YCWP produced from SCY that were published from January 2000 to December 2019 in peer-reviewed journals in English with full texts accessible. We searched three databases: Web of Science, PubMed and Scopus. The terms used for searching are “*Saccharomyces cerevisiae*”, “brewer’s yeast”, “baker’s yeast”, “yeast cell wall products”, “beta-glucan” or “mannan oligosaccharides”, with “milk production”, “milk yield” or “lactation”. Fifteen studies were identified with 10 studies on ruminants, 4 studies on sows and 1 study on rabbit does. Information on the study design and the results are provided in the Supplementary Table S1.

In 10 studies on ruminants, eight studies were supplementation studies using SCYS and/or YCWP in addition to the feed and concentrate [48,50,52,53,54,55,56,57], including two studies using both SCYS and YCWP [53,57], and a further two studies using SCYS as protein sources to replace soybeans [51,58]. Four of five studies supplementing with SCYS in ruminants found positive results on milk yields [48,50,52,57]. Studies using YCWP also had positive results. One study using MOS [54], two studies using beta-glucan [56,57] and another study using YCWP (containing MOS and beta-glucan) [52] found significant higher milk yields in supplementing groups compared to control groups. However, one study using YCWP (without information of composition of MOS and beta-glucan) found a nonsignificant increase in milk yield at an early lactation stage (day in milk < 120) [55].

The effect of SCYS on milk production was attributed to improving microflora metabolic activity [52], digestibility of feed and energy metabolism [48,55], improving mammary gland health shown as lower somatic cell count in milk [50,52,56,66], as well as improving immunity shown as increasing maternal blood gamma-globulin levels and other immunological parameters [48,50,57]. The researchers suggested that beta-glucan and MOS from SCYS were mainly responsible for improving maternal immunity and health status [48,52]. Beta-glucan is a natural immunomodulator influencing both humoral and cellular immunity in ruminants [56,67]. MOS can bind selected pathogenic microbes and prevent pathogen colonisation in the host’s gastrointestinal tract [48].

However, in studies that tested both SCYS and YCWP, the milk yield was higher in the SCYS groups than in the YCWP groups [52,57], which suggests that the benefit of yeast supplementation is not limited to the effect from beta-glucan and MOS.

The significant effect of SCYS on milk yield in ruminants may have been partly due to improving nutrition compared to the control group. Although the composition of feeds administered to study groups was identical, the supplementing studies provided limited information about the nutritional value of the SCYS used. Only one study reported the composition of the SCYS, which added an extra 4.3% crude protein into the diet of SCYS group [52]. Furthermore, there was limited information on how the feed and SCYS were consumed by the animals. Only one study reported to feed the SCYS “by hands” [53]. Thus, it is possible that ruminants in the supplement groups had better nutritional status compared with the animals in the control groups, if they consumed the same amount of feed in addition to the SCYS which provided significantly extra nutrition. The results from studies assessing the replacement of protein source with SCYS also support the above presumption. When SCYS replaced soybean meal as the protein source, providing similar nutrient concentration in both diets, no difference in milk yield or milk composition was observed in dairy goats [51,58].

In the literature, YCWP, but not SCYS, has been used to supplement lactating non-ruminants. Four studies that evaluate the effect of YCWP supplementation on sows and one study on rabbit does yield inconsistent results [62,63,64,65,68]. Litter weight was used as the outcome in these studies. Only two out of five studies found the litter weight of piglets and rabbits pups in the MOS group to be significantly higher at day 14 and day 18 of lactation [62] or at weaning [65]; another study found that the piglets’ body weight in beta-glucan groups was significantly higher at day 45 postpartum after weaning [63]. However, the piglets were also supplemented with beta-glucan from day 10 postpartum in this study, which may contribute to the significant weight gain after weaning [63]. The variation in doses and YCWP composition may have led to the inconsistent results among these studies. Moreover, the offspring usually started to consume feed in addition to milk a few days after birth, so the litter weight gain at weaning is a reflection of both milk consumption and feed utilisation. Since none of these studies evaluated the volume of milk yield, the results are weak and insufficient to indicate the effectiveness of supplementation on milk production in pigs and rabbits.

In addition to some evidence of a positive effect on milk production, there is also some evidence of SCYS impacting the milk composition in ruminants and non-ruminants. Supplementing with SCYS significantly increased milk fat in ruminants [53,57,58]. YCWP supplementation also significantly increased total protein in ewe milk [56] and sow milk [64]. SCYS also affected milk protein composition. The β-casein concentrations had a significant reduction and k-casein had a significant increase in ewe milk after 70 days supplementation with SCYS with a daily dose of 30 g/animal during lactation [69], and whey γ-globulin in sow milk significantly increased with supplementation of beta-glucan from gestation to lactation at the 200–300 ppm level [63]. Significant increases in milk IgG were also observed in two studies on sows supplemented with MOS with a daily dose of 8 g/animal at day 21 of lactation in one study [62] and a daily dose of 900 mg/feed at day 23 of lactation in another study [64].

The results from animal studies suggest that supplementation with SCYS has a strong positive effect on milk yield in ruminants, limited improvement on weight gain in suckling piglets and a possible effect on milk composition. Extrapolation of these results to humans is inappropriate because the proposed mechanism in animals may not be effective due to interspecies differences in digestion systems and physiology of lactation [70,71,72]. It is important to note that SCYS is commonly taken in the first few months of lactation in humans, but animal supplementation is usually conducted long-term, starting in gestation and continuing through the whole lactation period. Furthermore, the positive effect of SCYS on milk production in animals may come from the higher nutrition requirements required by multiple births, whereas humans predominantly have single births. Thus, the results from the animal studies only partially support the potential effectiveness of SCYS supplementation on increase in human milk production.

## 4. Hypotheses of the Mechanism of *Saccharomyces cerevisiae* Yeast-Based Supplement on Breast Milk Production

**Figure 1:**
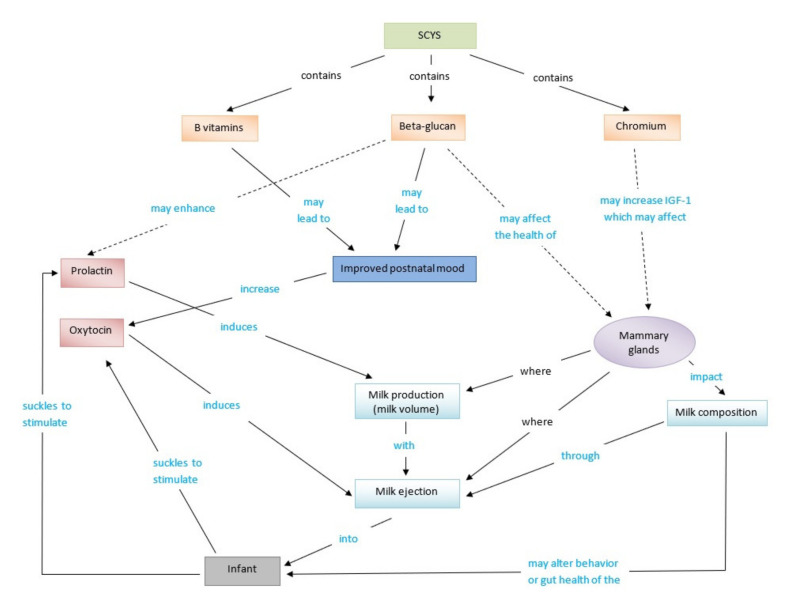
The potential mechanism of *Saccharomyces cerevisiae* yeast-based supplements (SCYSs) on breast milk production and composition. The dashed arrow means there is a lack of evidence in humans to support the proposed effect.

Although no scientific evidence directly supports the effectiveness of SCYS to influence breast milk production, hypotheses of the mechanism can be made based on the composition of SCYS, knowledge of physiology of human lactation and SCYS studies in lactating animals (Figure 1). We assume that SCYS may either increase the breast milk volume or improve women’s perception of milk production by changing milk composition and consequently influencing infant behaviour.

A literature review of Web of Science, PubMed and Scopus was conducted regarding each proposed mechanism. Studies in humans were primarily reviewed, but studies in animals and in vitro are also included when evidence from human studies was not found. The pathways of proposed mechanisms are described in the following sections.

First, high B vitamin content from SCYS supplementation may improve postnatal mood, leading to increased oxytocin release and milk ejection. SCYS is rich in B vitamins and minerals [73,74]. B vitamin deficiency is associated with negative mood changes, and B1, B6, folate, B12 or multi-nutrient supplementation is reported to improve symptoms in the general population [75,76]. Although there are limited data regarding B vitamin status and postnatal mood symptoms [77,78], one study showed that multi-nutrient supplementation containing several B vitamins and minerals had a better protective effect on postnatal depression than only calcium and vitamin D3 supplementation [79]. Consumption of yeast-based spreads, such as marmite and vegemite produced from SCY extract, has been reported to improve anxiety and stress symptoms but not depressive symptoms in the general population [80]. Supplementing with SCY-derived beta-glucan significantly reduced Profile of Mood States scores compared to placebo in non-lactating women with moderate stress [81]. Thus, taking SCYS during breastfeeding may improve postnatal mood symptoms such as stress, anxiety and depression.

Studies on postnatal distress and breast milk production suggested that the postnatal mood may be indirectly related to milk secretion by influencing oxytocin release [82,83]. Postnatal depression was associated with reduced length of exclusively breastfeeding period [84] and women with postnatal distress had higher risks of PIM [18,84]. However, no difference of breast milk volume was observed between women with and without perinatal depression [84]. A lower postnatal plasma oxytocin level was associated with greater postnatal mood symptoms [82,83], and psychological stress was reported to decrease suckling-induced pulsatile oxytocin release during one breastfeeding session [85,86,87]. A lower postnatal plasma oxytocin level was also inversely correlated to the baseline oxytocin before a breast feed in breastfeeding women [87]. Thus, improved postnatal mood may enhance oxytocin release and hence milk ejection. Improved milk ejection leads to better milk removal and may increase milk production. Improved milk ejection may also reduce infant frustration and allow infants to feed to demand. Therefore, increased oxytocin may reduce PIM.

Second, SCYS contains bioavailable chromium which may increase breast milk production by influencing insulin-like growth factor 1 (IGF-1). Chromium was reported to upregulate IGF-1 mRNA and IGF-1 receptor levels in rat skeletal muscle cells with presence of insulin [88]. However, the form of chromium supplemented in the cell culture in this study was different from the bioavailable chromium in SCYS. IFG-1 is a hormone found in both maternal blood and breast milk [89], which may benefit milk production in different ways. For example, higher levels of maternal blood IGF-1 can enhance mammary gland growth, improve blood flow and milk secretion [90], and breast milk IGF-1 can promote neonatal growth and nutrient absorption [90,91], and may optimise weight gain in exclusively breastfeeding infants [92,93].

Third, the large amount of beta-glucan and MOS in SCYS [43,94] may benefit milk quantity and quality through several pathways. Beta-glucan was reported to have a dose-related stimulation on the secretion of prolactin from GH3/B6 rat pituitary tumour cells [95]. SCY-derived beta-glucan has been reported to stimulate the innate immune system, for example, suppressing production of interferon-γ (IFN-γ) in vitro [96] and activating IL-1β transcription and secretion in human macrophages [97]. Supplementation of beta-glucan and MOS from SCYS or YCWP improves mammary gland health in lactating animals [50,52,56,66]. Moreover, orally taking SCY-derived beta-glucan has been reported to influence the synthesis and release of interleukins IL-6 and IL-10 in vivo [98,99,100] and reduce blood IL-6 and increase blood IL-10 in overweight and obese people [101]. These cytokines were also detected in breast milk and may potentially regulate infant gut immunity [102], although no maternal supplementation studies have evaluated human milk cytokines.

The other potential pathway through which SCYS could impact lactation is to affect infant demand by alteration in milk composition such as milk hormones and human milk oligosaccharides (HMOs). Researchers have suggested that SCY-derived beta-glucan supplementation could increase blood ghrelin in weanling piglets [103] and could lower blood leptin levels in patients with diabetic retinopathy [104], but there is no published information about the effect on milk composition. Human milk leptin and ghrelin could regulate infant breast milk intake by stimulating infant appetite [89]. However, the findings of studies on the relationship of human milk hormones and infant weight gain are inconsistent [105]. There is also a lack of studies that examine the relationship of milk hormones and milk intake [93].

Human milk oligosaccharides (HMOs) are the third most abundant component in human milk and benefits to infants are thought to include prebiotic effects, prevention of pathogen adhesion, modulation of intestinal epithelial cell responses and direct modulation on immune responses [106,107]. Very limited evidence suggests that maternal diet has an influence on milk HMOs’ abundance and profile [108,109]. This effect has been seen in rats, where high prebiotic fibre diets modified the amount of some milk oligosaccharides in rat and consequently influenced the establishment of gut microbiota in offspring [110].

Milk composition changes such as milk cytokines, milk hormones and HMOs may benefit infant growth and development as well as gut immunity and result in the improvement of colic, unsatisfied, unsettled or crying behaviour, which is commonly perceived by the mother as signs of insufficient milk production (PIM) [5,14,15,16]. Thus, although not directly influencing milk volume, SCYS could reduce PIM by influencing milk composition.

## 5. Safety Considerations of Taking *Saccharomyces cerevisiae* Yeast-Based Supplement during Lactation

SCYS is approved as a food additive by the USDA with total folic acid not exceeding 0.04 mg/g [35]. However, there is no regulation or recommendation on the safe dosage of SCYS in lactation. We searched Web of Science, PubMed and Scopus using “contamination”, “food-drug interaction” or “side effect” with “brewer’s yeast”, “nutritional yeast” or “*Saccharomyces cerevisiae*” to review the safety risks. Possible safety concerns from taking SCYS during breastfeeding are described as follows.

First, the nutrient contents vary in strains of SCY and batches of SCYS products [43]. This could increase the risk of nicotinic acid and folic acid intake approaching the upper level (UL) if women regularly consume a high dose of SCYS (i.e., 30 g/day as the highest dose recommended in Table 3) and take multivitamins or B vitamins supplements at the same time. The calculation of nicotinic acid and folic acid intake from SCYS and risk of approaching UL can be found in Appendix A.

Second, SCYS has been found to be contaminated with ochratoxin A (OTA) in Germany [111,112]. OTA is a type of mycotoxin [111] that can cause nephrotoxicity, immunotoxicity and carcinogenicity [113]. It can bind to yeast cell walls [114] and cause contamination of SCYS. OTA contamination is frequently reported in breast milk samples worldwide [115,116]. Consumption of breast milk contaminated with OTA may increase risks of renal injury in exclusively breastfeeding infants [117]. OTA is also a contaminant in many other foods including cereals, coffee, wine, grapes, meat and dairy foods [115,118]. Maternal blood OTA levels will increase if the mother habitually consumes contaminated foods, as the half-life in human blood is 35–36 days [115,116]. The OTA concentration in mature breast milk is about 8% to 44% of that in maternal blood [117,119]. A daily dose of 30 g of SCYS (the highest dose recommended in Table 3), along with dietary exposure, could result in an estimated OTA intake of 27.4% of Provisional Tolerable Weekly Intake (PTWI) for New Zealand lactating women. The estimation of OTA intake is described in Appendix A.

Third, SCYS may contain large amounts of tyramine that can interact with monoamine oxidase inhibitors (MAOIs). This interaction may cause a significant rise in blood pressure and increase the risk of heart attack or stroke [120,121]. As such, SCYS should be avoided when taking medications containing MAOIs.

Fourth, SCYS may aggravate inflammatory bowel diseases such as Crohn’s disease. Anti-*Saccharomyces cerevisiae* antibodies are suggested as biomarkers of Crohn’s disease [122]. One study found patients had significantly higher Crohn’s disease activity during time of exposure to baker’s yeast [123]. However, the authors did not say if the baker’s yeast was active or inactive, and not all patients had symptoms associated with yeast exposure. None the less, patients with Crohn’s disease need to be aware if taking SCYS.

Finally, minor adverse effects of taking SCYS were recorded in two [124,125] of seven [28,29,36,124,125,126,127] human studies, although none of these studies were conducted in lactating women. In one randomised placebo-controlled study on type 2 diabetes, one case of nausea was reported in the intervention group (*n* = 29) [125]. In another placebo-controlled crossover study on type 2 diabetes, one case of skin rash, one case of constipation, and one case of decreased appetite was documented in the intervention group (*n* = 14) [124]. In both studies, participants in the intervention group received chromium-enriched brewer’s yeast supplements.

There are four studies in which lactating women have been given SCYS (selenium enriched) for the purpose of evaluating maternal selenium supplementation on selenium concentration in breast milk [128,129,130,131]. Unfortunately, these studies did not report either the number of participants not completing the trial nor adverse effects from supplementation. Thus, the likelihood of adverse effects due to taking SCYS during lactation remains unknown.

## 6. Conclusions

SCYS is used as a galactagogue by breastfeeding women, but there is no peer-review evidence of its effectiveness and no recommendation or guideline of its use from any professional organisations. SCYS is available online with product names “brewer’s yeast” or “nutritional yeast”. The production process and nutrient composition vary between products. SCYS, in a range of doses, is recommended on the internet as a galactagogue to increase breast milk production. The inconsistent information may be misleading and cause confusion about use of SCYS as a galactagogue.

SCYS has been shown as a dietary supplement to increase milk production in ruminant animals. However, the influence of SCYS on breast milk production in humans has not yet been established. There are potential mechanisms through which postnatal supplementation of SCYS could increase breast milk volume or change milk composition, thus affecting infant behaviour and hence address PIM.

The safety of taking SCYS during lactation is unknown. It is approved by the USDA as a food additive and widely consumed as a supplement. However, minor adverse effects were reported in human supplementation studies with diabetics, and OTA contamination has been found in SCYS.

Further research is required to investigate the efficacy and safety of SCYS consumption as a galactagogue in breastfeeding women. Researchers should be aware that addressing milk volume as the endpoint is ethically difficult because women with true insufficient milk production need to increase milk production or supplement breastfeeding, i.e., they cannot rely on a randomised placebo-controlled trial. Researchers can test the mechanisms proposed in this review by investigating changes of milk composition, mother’s perception of milk production and maternal blood prolactin and oxytocin levels. The B vitamin concentrations and OTA concentration of SCYS should be determined before the trial to ensure intake is under the tolerable intake. Adverse effects should be monitored and recorded.

## Figures and Tables

**Table 1:** A selection of information on *Saccharomyces cerevisiae* yeast-based supplements (SCYSs) available on the internet *.

Product Name	Production Information	Dose (g/Day) **
Product 1: Brewer’s yeast powder ^1^	Species and strains: *Saccharomyces cerevisiae* Medium: unclear Other ***: “brewer’s yeast is generally from the fermentation of beer, adding grains (such as rice or wheat), malt, dried flowers of hops to the medium for cultivation”	30
Product 2: Brewer’s yeast powder ^2^	Species and strains: select strains of *Saccharomyces cerevisiae* Medium: sugar beet molasses	30
Product 3: Brewer’s yeast powder ^3^	Species and strains: *Saccharomyces cerevisiae* Medium: malted barley in the production of beer Other ***: “debittered”	15
Product 4: Nutritional yeast flakes ^4^	Species and strains: select strains of *Saccharomyces cerevisiae* Medium: enriched purified cane and beet molasses Other ***: “added niacin, pyridoxine hydrochloride, riboflavin, thiamin hydrochloride, folic acid and vitamin B12/ not from brewing process”	9
Product 5: Nutritional yeast flakes ^5^	Species and strains: *Saccharomyces cerevisiae* Medium: a mixture of sugar cane and beet molasses Other ***: “NOT brewer’s yeast, baker’s yeast or torula yeast”	15
Product 6: Nutritional yeast flakes ^6^	Species and strains: *Saccharomyces cerevisiae* Medium: molasses Other ***: “added niacin, pyridoxine HCl, riboflavin, thiamine HCl, folic acid and vitamin B12; gluten free”	20
Product 7: Brewer’s yeast powder ^7^	Species and strains: no information Medium: no information Other ***: “from brewing process”	11.5
Product 8: Brewer’s yeast powder ^8^	Species and strains: *Saccharomyces cerevisiae* Medium: no information Other ***: “from production of beer”	16
Product 9: Brewer’s yeast tablet ^9^	Species and strains: *Saccharomyces cerevisiae* Medium: no information Other ***: “manufactured using non-debittered brewer’s yeast powder”	1.8–3.6 (300 mg/tablet; 6–12 tablets/day)
Product 10: Brewer’s yeast tablet ^10^	Species and strains: no information Medium: no information	0.5–2 (500 mg/tablet; 1–4 tablets/day)

* Information accessed on 4 June 2020, from the advertisements of Google search of “brewer’s yeast”. ^**^ Doses were provided on the product package. *** Information quoted from the websites. ^1^ https://www.solgar.com/products/brewers-yeast-powder/, accessed on 4 June 2020. ^2^ https://www.bluebonnetnutrition.com/product/bluebonnet-nutrition-super-earth-brewers-yeast-powder-1-lb/, accessed on 4 June 2020. ^3^ https://nz.pipingrock.com/brewer-s-yeast/brewers-yeast-powder-debittered-100-pure-1-lb-454-g-9260, accessed on 4 June 2020. ^4^ https://www.nowfoods.com/supplements/nutritional-yeast-flakes, accessed on 4 June 2020. ^5^ https://foodsalive.com/products/nutritional-yeast-vegan-non-gmo, accessed on 4 June 2020. ^6^ https://www.luckyvitamin.com/p-1756-kal-nutritional-yeast-flakes-22-oz, accessed on 4 June 2020. ^7^ https://www.luckyvitamin.com/p-3420807-kal-brewer-s-yeast-powder-100-natural-unfortified-unsweetened-7-4-oz, accessed on 4 June 2020. ^8^ https://www.nowfoods.com/supplements/brewers-yeast-powder, accessed on 4 June 2020. ^9^ https://www.naturesaid.co.uk/brewers-yeast-300 mg.html, accessed on 4 June 2020. ^10^ https://www.thompsons.co.nz/products/general-wellbeing/brewers-yeast-tablets/, accessed on 4 June 2020.

**Table 2:** Description of B vitamin and mineral concentrations in a selection of commercially available *Saccharomyces cerevisiae* yeast-based supplements (SCYSs).

Nutrients	Content * µg/g Dry Yeast				
	Product 1 * Brewer’s Yeast Powder	Product 2 * Brewer’s Yeast Powder	Product 3 * Brewer’s Yeast Powder	Product 4 * Nutritional Yeast Flakes	Product 5 * Nutritional Yeast Flakes
Thiamin	10	30	20	666.7	1600
Riboflavin	30	60	60	666.7	5
Niacin	190	333.3	380	3555.6	1000
Vitamin B6	5	30	10	666.7	666.7
Vitamin B12	-	-	-	1.6	-
Folate (DFE)	11.4 ^**^	14.2	13.3	75 ^**^	-
Pantothenic acid	-	100	-	-	2333.3
Biotin	-	0.3	0.3	-	1
Calcium	-	1500	733.3	666.7	1733.3
Iron	20	40	40	55.6	466.7
Zinc	-	166.7	-	-	2000
Selenium	-	2.2	-	-	1.4
Chromium	-	0.43	-	-	0.3

DFE, dietary folate equivalent. * Product numbers match the numbers in Table 1. Nutrition information was accessed on 4 June 2020. ** On websites, the values were provided as folic acid; we calculated DFE as 1 µg DFE = 0.6 µg folic acid [39].

**Table 3:** A selection of information found on the internet about taking *Saccharomyces cerevisiae* yeast-based supplements (SCYS) to increase breast milk production *.

Author or Source	Product Information	Dose (g/Day)	Format of the Supplement	Ingestion Method	Claimed Benefits
Anne Smith, IBCLC ^1^	Brewer’s yeast	2.7 (300 mg tablet) or 4.5 (500 mg tablet) ^**^	Tablets	3 tablets taken with meals, 3 times per day	Increase milk production, contains B vitamins
Donna Murray, RN Reviewed by Meredith Shur, MD ^2^	Brewer’s yeast	No information	Tablets or powder	No information	Increase milk supply, improve mood and baby blues
Rohit Garoo, BSc. Reviewed by Briana Violand, IBCLC ^3^	Brewer’s yeast (used in brewing and making bread, but different from baker’s yeast)	30 g ***	Recommend using powder because the dose of tablets varies between manufacturers	Add to cookies or water, 3 tablespoons per day, can increase the quantity by half-a-teaspoon a day if not seeing any improvement	Anecdotally increases milk supply, improves acne, improves glucose tolerance in diabetes, considered as a nutritional supplement for B vitamins and selenium
Kelly Winder, doula ^4^	Brewer’s yeast (not substitutable with baker’s yeast or nutritional yeast)	Unclear ****	Powder or flakes	As an ingredient in lactation cookie recipe, 1 to 2 tablespoons per recipe, 2–5 cookies per day	Boost breast milk supply
Medela, breast pump manufacturer ^5^	Brewer’s yeast	Unclear ****	Powder	As an ingredient in lactation cookie recipe, 5 tablespoons per recipe, no information of how many cookies to take per day	Increase breast milk supply
Crystal Karges, RDN, IBCLC ^6^	Brewer’s yeast (can be substituted by nutritional yeast)	Unclear ****	Powder	As an ingredient in lactation cookie, 4 tablespoons per recipe, 2 cookies per day	Naturally help support milk supply, offer a boost of B vitamins, iron and other minerals

IBCLC, international board-certified lactation consultant. RN, registered nurse. MD, doctor in medicine. RDN, registered dietitian nutritionist. ^*^ Information accessed on 4 June 2020. ** Determined from 9 × 300 mg or 500 mg brewer’s yeast tablets in the Table 1, as no indication of brand or dose of brewer’s yeast tablets in this article. *** Determined by measuring 1 tablespoon (15 mL) brewer’s yeast powder, which weighs 10 g. **** Lactation cookie recipes without information on the cookie size or how many cookies per batch. ^1^ https://www.breastfeedingbasics.com/articles/increasing-your-milk-supply, accessed on 4 June 2020. ^2^ https://www.verywellfamily.com/foods-that-increase-breast-milk-supply-431598, accessed on 4 June 2020. ^3^ https://www.momjunction.com/articles/brewers-yeast-when-breastfeeding_00456918/, accessed on 4 June 2020. ^4^ https://www.bellybelly.com.au/breastfeeding/lactation-cookies/, accessed on 4 June 2020. ^5^ https://www.medelabreastfeedingus.com/article/298/oatmeal-chocolate-chip-lactation-cookies, accessed on 4 June 2020. ^6^ https://www.crystalkarges.com/blog/family-friendly-lactation-oat-cookie-recipe, accessed on 4 June 2020.

## Supplementary Materials

The following are available online at https://www.mdpi.com/2072-6643/13/3/727/s1, Table S1: Effectiveness of *Saccharomyces cerevisiae* yeast-based supplement (SCYS) on milk production in animals.

## Appendix A

The Australia/New Zealand Nutrient Reference Values (NRV) of nicotinic acid and folic acid are used for comparison the risk of approaching the upper level (UL) of each vitamin [39]. The upper level (UL) of nicotinic acid for lactation is set at 35 mg/day for women aged 19 and above, and folic acid from fortified foods or supplement for lactation is set at 1 mg/day for women aged 19 and above.

The calculation on nicotinic acid intake from SCYS is based on the following assumptions:

1. A breastfeeding woman (>18 years) consumes 30 g/day SCYS;
2. The concentration of nicotinic acid in SCYS is 1 mg/g, as per the highest value in Ahmad and Moat’s study of SCY grown in culture added with 500 µg/mL tryptophan [132].

The estimated nicotinic acid intake is 1 mg/g × 30 g/day = 30 mg/day, which is 85.7% of UL.

The calculation on folic acid intake from SCYS is based on the following assumptions:

1. A breastfeeding woman (>18 years) consumes 30 g/day SCYS;
2. The concentration of folic acid in SCYS is 0.045 mg/g, as per the highest value in Table 2 (75 µg DFE = 45 µg folic acid).

The estimated folic acid intake is 0.045 mg/g × 30 g/day = 1.35 mg/day, which is 135% of UL.

The calculation of Ochratoxin A (OTA) intake is based on the following assumptions:

1. A breastfeeding woman (>18 years) consumes 30 g/day SCYS;
2. The SCYS contains 4.24 ng/g OTA as the highest level reported in Germany study [112];
3. The body weight of the woman is 70 kg;
4. The consumption of OTA from other source is 2.1 ng/kg body weight/day as the highest OTA dietary exposure for New Zealand women [133].

The estimated OTA intake is 4.24 ng/g × 30 g/day × 7 days ÷ 70 kg body weight + 2.1 ng/kg body weight/day × 7 days = 27.42 ng/kg body weight/week.

We used the Provisional Tolerable Weekly Intake (PTWI) of OTA (100 ng/kg body weight/week) assessed by the FAO/WHO Expert Committee on Food Additives (JECFA) to do the calculation [134]. The estimated OTA intake is 27.4% of PTWI.
