# Supplementary material for: Saccharomyces cerevisiae Yeast-Based Supplementation as a Galactagogue in Breastfeeding Women? A Review of Evidence from Animal and Human Studies
Source: Nutrients. 2021 Feb 25;13(3):727. doi: 10.3390/nu13030727 (PMC7996189; doi:10.3390/nu13030727)
Supplement: Supplementary file 1 [file nutrients-13-00727-s001.pdf]

Table S1 Effectiveness of *Saccharomyces cerevisiae* yeast-based supplement (SCYS) on milk production in animals.

References	Study animal	Study design	Daily dose	Administration	Daily milk yields or litter weight	Other benefits
Ruminant animals						
Dobicki et al. [1]	Cow, Holstein-Friesian N = 75	No information. Daily milk yield were determined once a month at the Milk Analysis Laboratory.	*SCYS: 200 g/animal *YCWP: 20 g/animal	Supplementation from 7 days prior to the estimated calving date to 100 days of lactation. SCYS group: dried yeast (Leiber BT) containing 40% yeast cells. YCWP group: MOS (Biolex MB 40) containing 40-50% MOS and beta-glucans.	Milk yields at 100 day: SCYS: 26.780 ± 4.459^{A} kg MOS: 25.993 ± 5.707^{A} kg Control: 23.810 ± 4.765^{B} kg ^{A,B} P≤0.01	Milk SCC was significantly lower (P≤0.01) in SCYS group. The number of bacteria in the rumen fluid was significantly higher (P≤0.01) in SCYS and MOS groups (n = 24).
Kuczaj et al. **[2]**	Cow, Holstein-Friesian (Red-white) N = 50	Animals randomly assigned to 2 groups. Daily milk yield was conducted once a month (official control).	*SCYS: 200 g/animal	Supplementation from 3 weeks before calving to 100 days lactation with added SCYS (Leiber BT) containing 40% yeast cells.	Milk yields at 100 day: SCYS: 29.84 ± 4.80 kg Control: 28.60 ± 4.82 kg P> 0.05	Milk fat and dry matter were significantly higher (P< 0.05) in SCYS group in the first month. Maternal blood urea was significantly higher (P≤0.01) and blood albumins and globulins were significantly lower (P≤0.01 and ≤0.05 respectively) in SCYS group (n = 16). Milk SCC was significantly lower (P<0.05) in SCYS group in the first two months.
Westland et al. **[3]**	Cow, Holstein cross Friesian N = 80	80 cows randomly assigned to 2 groups prepartum with colostrum obtained from 59 cows. colostrum collected by Fullwood® mobile milking	*MOS: 2 g/animal	Supplementation prepartum from drying off until point of calving over 6 months. No information of the MOS.	Colostrum yields immediately post-calving: MOS: 7.5 ± 0.69 kg Control: 5.6 ± 0.43 kg P = 0.02	No significant differences of IgG in colostrum between two groups.

		machine (Fullwood Ltd, Ellesmere, Shropshire, UK) within 40 minutes of calving.				
Aung et al. **[4]**	Cow, Holstein<br>N = 32	2×2 factorial arrangement (2 treatments × 2 LP)<br>LP = lactation period, early lactation means day in milk <120, and mid lactation means >120<br>Cows were milked twice a day and milk yields were automatically recorded at each milking.	*YCWP:<br>10 g/animal	Ten-week experiment with first 2 weeks as adaptation period and last 8 weeks as experimental period, with added YCWP (SafMannan; Phileo, Lesaffre Animal Care, France)	Milk yields:<br>Control:<br>Early lactation: 25.52±1.96 kg<br>Mid lactation: 24.48±1.73 kg<br>YCWP:<br>Early lactation: 28.97±1.54 kg<br>Mid lactation: 22.83±1.82<br>P = 0.627 (treatment)<br>P = 0.117 (treatment × LP)	In early lactation groups, maternal blood total cholesterol and non-esterified fatty acid/total cholesterol ratio was significantly lower in treatment group.<br>In early lactation groups, 4% fat corrected milk and energy<br>corrected milk was higher (p = 0.063 and 0.065 respectively) in treatment group.
Milewski & Sobiec **[5]**	Ewe, Polish long-wool Kamieniec<br>N = 26	Animals divided into 2 equal groups. No information of randomisition and blind assessment.<br>Daily milk yield was determined by the results of milking after	*SCYS:<br>30 g/animal	Supplementation for 70 days lactation period after birth, with added SCYS (Inter Yeast®, Krośniewice).	Milk yields:<br>Day 28:<br>SCYS: 1895.00 ml<br>Control: 1603.31 ml<br>P< 0.01<br>Day 70:<br>SCYS: 1121.54 ml<br>Control: 970.77 ml<br>P< 0.05	On day 28 blood erythrocytes and leukocytes, blood glucose and Na+ (P≤0.05), blood haemoglobin and haematocrit and blood Cl- (P≤0.01) were significantly higher and blood creatinine was significantly lower (P≤0.01) in SCYS group.<br>On day 70 blood leukocyte, blood glucose and Na+ (P≤0.05), blood Cl- (P≤0.01) were significantly higher and blood creatinine was significantly lower (P≤0.01) in SCYS group.

		an injection of oxytocin.				
Zabek et al. **[6]**	Ewe, Polish long-wool Kamieniec N = 26	Animals divided into 2 equal groups. No information of randomisition and blind assessment. No information of milking.	*Beta-glucan: 0.18g/animal (adding beta-glucan as 3 g/kg of CJ mixture at the daily dose of 0.6 kg/animal). Assume feeding at equal level.	Supplementation from birth to 70 days of lactation with added beta-glucan (Biolex®-Beta S Leiber GmbH) containing 70% of beta-1,3/1,6-D-glucan not changing the chemical composition of the feed.	Milk yields:<br>Day 28:<br>Control: 1596.15 ± 181.90 ml<br>Glucan: 1812.31 ± 231.31 ml<br>P< 0.01<br>Day 70:<br>Control: 998.46 ± 187.69 ml<br>Glucan: 1138.69 ± 155.46 ml<br>P< 0.01	Milk SCC was significantly lower (P≤0.05) in glucan group on day 28. Milk fat was significantly higher (P≤0.01) in glucan group on both day 28 and day 70. Milk protein was significantly higher (P≤0.05) in glucan group on day 70.<br>Blood gamma globulin, lysozyme activity, respiratory burst activity, potential killing activity, MTT-ConA (RI) and MTT- LPS (RI) were significantly higher (p≤0.01) in glucan group on both day 28 and day 70.
Zabek et al. [7]	Ewes, Kamieniec N = 39	Animals divided into 3 equal groups. No information of randomisition and blind assessment. Daily milk yield was determined by the results of morning milking after an injection of oxytocin.	*SCYS: 15 g/animal	Experimental group I supplementation from late pregnancy to 70 days of lactation, experimental group II supplementation from birth to 70 days of lactation. Supplementation with SCYS (Inter Yeast S).	Milk yields:<br>Day 28<br>Group I: 1868.46±268.88[a] ml<br>Group II: 1927.54±439.58[a] ml<br>Control: 1603±164.89[b] ml<br>Day 70<br>Group I: 1078.46±168.22 ml<br>Group II: 1164.61±286.84[a] ml<br>Control: 970.77±158.87[b] ml<br>[a,b] P<0.05	Milk SCC was significantly lower in supplementation groups on day 28 and day 70 (P≤0.01 and ≤0.05 respectively).<br>Higher value of blood gamma globulin, lysozyme activity, respiratory burst activity, MTT-ConA (RI) and MTT- LPS (RI) were observed in group I on both day 28 and day 70, compared to the other two groups (P≤0.01). Ceruloplasmin activity and potential killing activity in group I were significantly higher (P≤0.01) than control on both days. All these parameters except for gamma globulin in group II were significantly higher (P≤0.01) than control on both days.
Zabek et al. [7]	Ewes, Kamieniec N = 26	Animals divided into 2 equal groups. No information of randomisition	*SCYS: 15 g/animal	Supplementation from birth to 70 days of lactation with added SCYS (Inter Yeast S).	Milk yields:<br>Day 28<br>SCYS: 1860.15±320.46 ml<br>Control: 1596.15±181.90 ml<br>P<0.05<br>Day 70<br>SCYS: 1277.92±175.47 ml	Milk SCC was significantly lower (P≤0.05) in SCYS group on day 28.<br>Blood gamma globulin, lysozyme activity and MTT-ConA (RI) (P≤0.01) as well as respiratory burst activity, potential killing activity and MTT- LPS (RI) (p≤0.05) were significantly higher in SCYS group on day 28.

		and blind assessment. Daily milk yield was determined by the results of morning milking after an injection of oxytocin.			Control: 998.46±187.69 ml P<0.01	All these 6 parameters were significantly higher (P≤0.01) in SCYS group on day 70.
Zaleska et al. [8]	Ewe, Polish long-wool N = 120	Animals divided into 3 equal groups. Milk yield evaluated from 8 randomly selected ewes nursing singleton in each group. Daily milk yield was determined by the results of morning milking after an injection of oxytocin.	*SCYS: 50 g/kg feed Beta-glucan: 3 g/kg feed	Supplementation 3-week preparation for tupping + from lambing to 70 days lactation. SCYS group: dried yeast (Inter Yeast®) Glucan group (G): beta-glucan (Biolex® Beta-S) containing over 70% (1,3)-(1,6)-β-D-glucan.	Milk yields: Day 28 SCYS: $1850.00{\pm}231.02^{a}$ ml G: $1795.00{\pm}211.59^{a}$ ml Control: $1567.50{\pm}172.69^{b}$ ml Day 70 SCYS: $1373.50{\pm}212.86^{A}$ ml G: $1268.50{\pm}70.05^{A}$ ml Control: $1055.00{\pm}155.56^{B}$ ml ^{a,b} P<0.05, ^{A,B} P<0.01	On day 28 milk fat and dry matter were significantly higher (P≤0.01) in glucan group and milk dry matter was significantly higher(P≤0.01) in SCYS group, compared to the control group. On day 70 milk fat and dry matter were significantly higher (P≤0.01 and ≤0.05 respectively) in glucan group and milk fat was significantly higher(P≤0.05) in SCYS group, compared to the control group.

Gomes et al. [9]	Goat, Saanen N = 24	Animals randomly assigned to 3 × 2 factorial design (3 diets × 2 parity orders). Goats were milked manually twice a day and milk yields of individual goat was measured at each milking.	**SCYS group: 234.1 g/kg feed SCYS plus soybean meal group (SCYS+S): 97.2 g/kg feed Goats were fed *ad libitum*.	SCYS in substitution to soybean meal. Diet in SCYS groups provided the same crude protein in dry matter as in soybean meal group (S). The experiment was conducted from 21 days to 200 days of lactation. No information of source of yeast.	Milk yields (an average of daily milking): 21 – 60 days: SCYS: 3.06±0.23 kg SCYS+S: 2.94±0.23 kg S: 3.21±0.23 kg 60 – 130 days: SCYS: 2.88±0.37 kg SCYS+S: 2.79±0.37 kg S: 3.75±0.37 kg 130 – 200 days: SCYS: 2.71±0.24 kg SCYS+S: 2.69±0.24 kg S: 3.34±0.24 kg No significant difference.	
De Lima et al. [10]	Goat, Saanen N = 18	Completely randomized design. Goats were milked twice a day and milk yields of individual goat was recorded at each milking.	**SCYS group: 229.0 g/kg feed SCYS plus soybean meal group (SCYS+S): 102.0 g/kg feed Goats were fed *ad libitum*.	SCYS in substitution to soybean meal. Diet in SCYS groups provided the same crude protein in dry matter as in soybean meal group (S). The experiment was conducted from 60 days to 150 days of lactation. Yeast grown on sugar cane.	Milk yields: SCYS: 2.2 kg SCYS+S: 2.3 kg S: 2.3 kg P = 0.923	Milk produced by SCYS+S group contained significantly higher fat and total solids than SCYS group (P<0.05). Milk production efficiency (kg of milk produced/kg of crude protein ingested) was significantly higher in SCYS group compared to SCYS+S and S groups (P<0.05).
Pigs						

Czech et al. [11]	Sow, Polish Landrace breed<br>N = 32 in Experiment 1 and Experiment 2 respectively	No information.	*MOS:<br>8 g/animal	Supplementation 4 weeks prepartum and 4 weeks postpartum with added MOS (Bio-MOS, Alltech, USA).<br>The diet in Experiment 1 contained 40% wheat; Experiment 2 contained 40% triticale.	Litter weight in Experiment 1<br>At birth:<br>MOS: 22.29 kg<br>Control: 19.43 kg<br>P = 0.032<br>Day 14:<br>MOS: 59.67 kg<br>Control: 48.17 kg<br>P = 0.016<br>Day 28:<br>MOS: 103.77 kg<br>Control: 84.69 kg<br>P = 0.013<br>No difference in Experiment 2	In both experiments, MOS groups had significantly lower piglet losses compared to the control groups (P≤0.05).<br>In Experiment 1, MOS group had significantly higher contents of colostrum IgG and IgM from 1 hour to 48 hours after birth (P≤0.05).<br>In Experiment 2, MOS group had significantly higher content of colostrum IgG from 1 hour to 48 hours after birth (P≤0.05), and significantly higher content of colostrum IgM from 1 hour to 12 hours after birth (P≤0.05).<br>In both experiments, MOS group had significantly higher content of milk IgG at 21st day of age (P≤0.05).
Szuba-Trznadel et al. [12]	Sow, crossbred (Polish Large White × Polish Landrace)<br>N = 40	No information.	*Beta-glucan:<br>Experimental group I: 100 ppm feed,<br>Experimental group II: 200 ppm feed,<br>Experimental group III: 300 ppm feed	Supplementation from day 80 of gestation to day 21 of lactation (weaning) with added purified (1,3)-(1,6)-β-D-glucan (Betamune®, 80% purity).<br>Piglets started supplementation from day 10 of lactation to the end of experiment with the same dose as sows.	Piglet body weight<br>Day 2:<br>Experimental I: 1.64 ± 0.23 kg<br>Experimental II: 1.66 ± 0.12 kg<br>Experimental III: 1.63 ± 0.12 kg<br>Control: 1.58 ± 0.25 kg<br>No significant difference.<br>Day 21:<br>Experimental I: 7.20 ± 0.70 kg<br>Experimental II: 6.98 ± 0.70 kg<br>Experimental III: 6.95 ± 0.67 kg Control: 6.73 ± 0.80 kg<br>No significant difference.<br>Day 45:<br>Experimental I: 14.63 ± 0.58 kg<br>Experimental II: 15.11 ± 0.62[b] kg<br>Experimental III: 15.05 ± 0.60[b] kg	Milk whey γ-globulin was significantly higher (p<0.01) in Experimental I and Experimental II groups compared to control group on day 21.<br>Sow serum α-globulin was significantly higher (p<0.01) in Experimental II group compared to control group on day 2.<br>Sow serum γ-globulin was significantly higher (p<0.01) in Experimental II group compared to control group on day 21.<br>Piglet serum α-globulin was significantly higher (p<0.05) in all Experimental groups compared to control group on day 2.<br>Piglets in experimental groups had significantly lower (p<0.01) feed conversion ratio from day 21 to day 45 compared to control group.

					Control: 14.27 ± 0.62[a] kg<br>[a,b] P<0.05	
Graugnard et al. [13]	Sow, no information of breed<br>N = 218	Randomized complete block design<br>62 milk samples collected from individual sows from week 1 to week 4, 22 from control and 40 from treatment group	*MOS:<br>900 mg/kg feed	Supplementation from day 100 of gestation to day 23 of lactation (weaning) with added MOS (Actigen™, Alltech Inc., Nicholasville, KY, USA)	Litter weight<br>At birth:<br>MOS: 19.7 kg<br>Control: 19.1 kg<br>P = 0.2<br>Adjusted wean weight<br>MOS: 7.0 kg<br>Control: 7.0 kg<br>P = 1.0	Milk protein, total solids less fat and IgG were significantly higher (p = 0.01, p = 0.03 and p = 0.03 respectively) in MOS group.<br>Intestinal gene expression in piglets from MOS group shown significantly higher (p<0.01) number of issue development, cell proliferation, cell growth and cell differentiation, as well as significantly lower (p<0.01) cell migration.
Duan et al. [14]	Sow, crossbred (Large White × Yorkshire, 4 ± 1 parity)<br>N = 60	Completely randomized design with a 2 × 2 factorial treatment arrangement (2 treatment × 2 animal groups). Colostrum and milk samples were collected from 6 sows per group.	*MOS:<br>Sow diet: 400 mg/kg feed<br>Piglet diet: 800 mg/kg feed	Supplementation from day 86 of gestation to day 20 of lactation (weaning) with added MOS (Actigen, Alltech Inc., Nicholasville, USA).<br>Piglets started supplementation from day 7 of lactation to the end of experiment with the same MOS.	Piglet body weight<br>At birth:<br>MOS: 1.56 kg<br>Control: 1.57 kg<br>P = 0.86<br>Weaning:<br>MOS: 6.10 kg<br>Control: 5.69 kg<br>P = 0.03	Piglet serum IgA and IgG concentrations at weaning were significantly higher (p<0.01) in group of sow diet supplementing MOS.<br>Piglet serum complement 3, complement 4 and lysozyme were significantly higher (p<0.01, p = 0.05 and p<0.01 respectively) in group of sow diet supplementing MOS.
Other animals						

Wu et. al [15]	Doe, New Zealand White N = 30	Completely randomized design.	*Beta-glucan: Experimental group I: 0.064% of feed Experimental group II: 0.128% of feed	Supplementation from day 14 of gestation to day 28 of lactation (weaning) with added (1,3)-(1,6)-β-D-glucan (Allamond, Inc.) containing 16% glucan.	Litter weight:<br>At birth:<br>Experimental I: 364 ± 32 g<br>Experimental II: 296 ± 46 g<br>Control: 319 ± 41 g<br>At weaning:<br>Experimental I: 2245 ± 334 g<br>Experimental II: 1960 ± 409 g<br>Control: 2205 ± 421 g<br>No significant difference.	Doe serum IgM was significantly ($p<0.05$) higher in experimental group I on lactation day 28. Does serum IgG was significantly ($p<0.05$) higher in experimental group II on lactation day 3 and significantly ($p<0.05$) higher in both experimental groups on lactation day 28.

MOS: manna oligosaccharides, SCC: somatic cell count, YCWP: yeast cell wall product.

* Added in addition to the feeds.

** Added as the substitute for soybeans as protein source.

References:

1. Dobicki, A.; Preś, J.; Zachwieja, A.; Kwaśnicki, R. *Saccharomyces cerevisiae* preparations in the feeding of cows and their effect on milk yield and composition as well as rumen microorganisms. *Electron J Pol Agric Univ* **2006**, *9*, 48.
2. Kuczaj, M.; Dobicki, A.; Pres, J.; Zachwieja, A.; Jakus, W. An influence of dried brewer's yeast (*Saccharomyces cerevisiae*) addition before and after calving on yield and chemical composition of milk and biochemical indices of blood in the first 100 days of lactation. *Electron J Pol Agric Univ* **2010**, *13*, 12-art 12.
3. Westland, A.; Martin, R.; White, R.; Martin, J.H. Mannan oligosaccharide prepartum supplementation: effects on dairy cow colostrum quality and quantity. *Animal* **2017**, *11*, 1779-1782.
4. Aung, M.; Ohtsuka, H.; Izumi, K. Effect of yeast cell wall supplementation on production performances and blood biochemical indices of dairy cows in different lactation periods. *Vet World* **2019**, *12*, 6, 796-801.
5. Milewski, S.; Sobiec, P. Effect of dietary supplementation with *Saccharomyces cerevisiae* dried yeast on milk yield, blood biochemical and haematological indices in ewes. *B Vet I Pulawy* **2009**, *53*, 753-758.
6. Ząbek, K.; Milewski, S.; Wójcik, R.; Siwicki, A.K. Effect of β-1, 3/1, 6-D-glucan in diet on productivity and humoral and cellular defense mechanisms in sheep. *Acta Vet Brno* **2013**, *82*, 141-146.
7. Zabek, K.; Milewski, S.; Wojcik, R.; Siwicki, A.K. The effects of supplementing diets fed to pregnant and lactating ewes with *Saccharomyces cerevisiae* dried yeast. *Turk J Vet Anim Sci* **2014**, *38*, 200-206.
8. Zaleska, B.; Milewski, S.; Ząbek, K. Impact of *Saccharomyces cerevisiae* supplementation on reproductive performance, milk yield in ewes and offspring growth. *Arch Anim Breed* **2015**, *58*, 79-83.
9. Gomes, L.C.; Alcalde, C.R.; de Macedo, F.D.F.; dos Santos, G.T.; Valloto, A.A.; de Lima, L.S.; Molina, B.S.D. Performance of lactating goats fed diets containing inactive dry yeast. *Rev Bras Zootec J Anim Sci* **2012**, *41*, 2249-2254.
10. de Lima, L.S.; Alcalde, C.R.; Freitas, H.S.; de Labio Molina, B.S.; Fonseca de Macedo, F.d.A.; Horst, J.A. Performance of dairy goats fed diets with dry yeast from sugar cane as protein source. *Rev Bras Zootec J Anim Sci* **2012**, *41*, 232-236.
11. Czech, A.; Grela, E.R.; Mokrzycka, A.; Pejsak, Z. Efficacy of mannanoligosaccharides additive to sows diets on colostrum, blood immunoglobulin content and production parameters of piglets. *Pol J Vet Sci* **2010**, *13*, 525-531.
12. Szuba-Trznadel, A.; Rzasa, A.; Lira, R.; Fuchs, B. The influence of (1,3)-(1,6)-beta-D-glucan on the production results of sows and their offspring. *J Anim Feed Sci* **2014**, *23*, 228-235.
13. Graugnard, D.E.; Samuel, R.S.; Xiao, R.; Spangler, L.F.; Brennan, K.M. Intestinal gene expression profiles of piglets benefit from maternal supplementation with a yeast mannan-rich fraction during gestation and lactation. *Animal* **2015**, *9*, 622-628.

14. Duan, X.D.; Chen, D.W.; Zheng, P.; Tian, G.; Wang, J.P.; Mao, X.B.; Yu, J.; He, J.; Li, B.; Huang, Z.Q., et al. Effects of dietary mannan oligosaccharide supplementation on performance and immune response of sows and their offspring. *Anim Feed Sci Tech* **2016**, *218*, 17-25.
15. Wu, H.H.; Weng, B.B.C.; Chen, K.L.; Chiou, P.W.S.; Yu, B. Effect of dietary supplementation of beta-1,3-1,6-glucan on reproductive performance and immunity of New Zealand White does and their pups. *Livest Sci* **2011**, *135*, 70-75.
